# St. Bartholomew's Hospital

**Published:** 1903-08-01

**Authors:** Marcus Samuel

**Affiliations:** Lord Mayor, Chairman for and on behalf of the Committee


### HOSPITAL ADMINISTRATION.

### CONSTRUCTION AND ECONOMICS.

### ST. BARTHOLOMEW'S HOSPITAL.

REPORT OF THE MANSION HOUSE COMMITTEE.

We give below the report of the Mansion House Committee. This report is disappointing, unsatisfactory, inadequate, and incomplete. It is disappointing because the committee had the most splendid opportunity which any body of men or any Lord Mayor has ever had in recent times to do something really effective and great for the City of London, which they have missed. It is unsatisfactory because it represents neither the views, wishes, nor opinions of the great medical staff of St. Bartholomew's Hospital, nor of the intelligent opinion in regard to modern hospital construction of the country at the present time. It is inadequate in the sense that it makes no provision for modern hospital buildings which are urgently called for, and recommends apparently no action on the part of the governors, except possibly the building of a new out-patient department, until sufficient funds are collected. It is incomplete because the summary of the evidence appended to the report is distinctly misleading in substance and in fact. We feel it to be a public duty to endeavour to save St. Bartholomew's Hospital from the injury which must result to that institution should the reconstruction of the hospital buildings be attempted on the lines apparently proposed by the committee. It is certain that matters cannot be allowed to rest so far as St. Bartholomew's Hospital is concerned where they are left by the Mansion House Committee's report, a careful reading of which gives the impression that the committee themselves were hopelessly divided on so many points that nothing but a fogged and indefinite report was possible.

REPORT OF THE MANSION HOUSE COMMITTEE.

At a meeting of the "Appeal Committee" of the Governors of St. Bartholomew's Hospital, held at the Mansion House on January 19th, 1903, the Right Hon. the Lord Mayor being in the chair, the following resolution was passed:—

"That having regard to the criticism upon the proposed appeal for the enlargement of St. Bartholomew's Hospital, based on inaccurate information, a committee be appointed to report—

1. "Whether it is desirable in the public interest and on financial grounds to retain St. Bartholomew's Hospital on its present site;

2. "In the event of the retention of the hospital on its present site, whether any better scheme of rebuilding than that suggested by the Governors can be devised.

3. "Upon any other matters affecting the hospital that the committee may think it desirable to inquire into.

"That such committee do consist of 15 members, nine to be nominated by the Lord Mayor and six by the treasurer of the hospital, and that the Lord Mayor and the treasurer be *ex officio* members of the committee."

In accordance with this resolution the following nine gentlemen were nominated by the Right Hon. the Lord Mayor, viz.:—The Right Hon. Lord Sandhurst, G.C.S.I., G.C.I.E., Chairman of the Middlesex Hospital, late Governor of Bombay; the Right Hon. Sir William Hart Dyke, Bart., M.P.; the Right Hon. Sir Savile Crossley, Bart., M.P., Honorary Secretary of King Edward's Hospital Fund; Sir Thomas Jackson, Bart., formerly Chief Manager of the Hong Kong and Shanghai Bank; Sir William Emerson, Past President of the Royal Institute of British Architects; Dr. Pye-Smith, F.R.S., Vice-Chancellor of London University, Consulting Physician to Guy's Hospital; the Hon. Alban Gibbs, M.P.; Mr. Richard Biddulph Martin, M.P.; Mr. Arthur Hill.

Sir Trevor Lawrence, as treasurer of the hospital, nominated:—Mr. Alderman Alliston; Sir William Church, Bart, K.C.B., President of the Royal College of Physicians; Mr. Benjamin L. Cohen, M.P.; Mr. Frederick Morris Fry, a member of the committee of King Edward's Hospital Fund; Mr. John Cary Lovell; Alderman Sir William P. Treloar.

The Lord Mayor, as chairman, appointed Mr. Graham Tahourdin to act as secretary to the committee.

The committee proceeded to examine the questions submitted to them, and beg to report:—

1. *As to Hospital Site.*—The committee investigated this point very fully. They received and examined a large amount of evidence given both by inhabitants of the neighbourhood and by others, and they came to the conclusion, with only one dissentient, that it was impossible in the public interest to entertain the idea of removing St. Bartholomew's Hospital from its present site.

In this connection they went thoroughly into the question of St. Luke's site, and came to the conclusion that such a removal, even were the site available, would not give the results its advocates anticipate, and that on that site the hospital would be cramped for room and not able to perform its duties even as efficiently as at present.

No evidence was brought forward in support of this plan that commended itself to the committee.

The committee proceeded to investigate, with great care and with the assistance of evidence from competent persons, the value of the present site of the hospital. They came to the conclusion that the value of the site of St. Bartholomew's Hospital has been much exaggerated, and that there would be very little, if any, ultimate money profit to the hospital in removing the building from its present situation to any other locality.

2. *As to Buildings*:—The Committee next carefully considered the present buildings of the hospital, and the necessary additions demanded by the medical and surgical staff.

They are satisfied that important additions to and a considerable re-arrangement and improvement of the existing buildings are necessary, and they consider that with the additional land purchased from Christ's Hospital, there will be ample room for the provision of a hospital with every modern appliance.

The Committee, having carefully examined the several plans placed before them, consider that a thoroughly efficient hospital can be provided by a gradual building scheme under which the improvements and alterations contemplated can be secured so soon as funds are obtained.

The committee are assured of the great value of the medical school to the hospital and the public. For the continued efficiency of the treatment of the patients, the teaching in the school and the advancement of medical science, greater facilities for research and teaching are absolutely necessary and call for additional accommodation.

3. The committee also carefully examined the financial position of the hospital itself. The evidence showed that its properties and revenues are judiciously and economically administered, and that the cost per bed compares favourably with that of other hospitals. They find that it is impossible to hope that funds for the additions to and rearrangement of the hospital can be obtained from the resources of the hospital itself. Indeed, the recent purchase of land from Christ's Hospital will entail a charge of over £9,000 per annum on its present revenue, leaving a deficit of £7,000 per annum on the ordinary expenditure.

The Committee recommend that the rebuilding of such parts of the hospital as are most urgently needed be proceeded with so soon as sufficient funds are collected. They consider that the Governors of St. Bartholomew's Hospital are fully justified in appealing to the public for assistance, and they heartily commend this appeal to the consideration of the citizens of London and to the public generally.

The Committee do not think that they would be justified in concluding their functions without placing upon record their opinion that from the evidence brought before them the administration of the hospital has been conducted by the Governors in a wise and enlightened spirit, with a due regard to economy, and in the best interests of the patients.

Marcus Samuel, Lord Mayor,
Chairman for and on behalf of the Committee.

July 27th, 1903.

